# Long-term results of huge deep-seated liposarcoma in the thigh: Two case reports

**DOI:** 10.1097/MD.0000000000033753

**Published:** 2023-05-17

**Authors:** Hyung Woo Wang, Youn Hwan Kim, Seong Oh Park

**Affiliations:** a Department of Plastic and Reconstructive Surgery, Hanyang University College of Medicine, Seoul, Republic of Korea.

**Keywords:** liposarcoma, marginal excision, well-differentiated liposarcoma

## Abstract

Liposarcoma is an uncommon malignant tumor that develops in the fatty tissue; thus, the long-term follow-up results of extremely large liposarcomas in the submuscular layer of the thigh have rarely been reported. Here we share the course and outcome of 2 cases of a huge deep-seated liposarcoma in the thigh.

**Patient concerns:**

Two patients visited our clinic, each with a deep-seated mass in the thigh. First, a 44-year-old man presented to the outpatient clinic with a left thigh mass. Approximately 1 year later, an 80-year-old man presented to the outpatient clinic with a right posterior thigh mass.

**Diagnosis:**

Magnetic resonance imaging revealed an approximately 14 × 8 × 21 cm well-differentiated liposarcoma between the sartorius and iliopsoas muscle and an approximately 14 × 12 × 31.5 cm lipomatous mass in the posterior compartment of the right thigh involving the right adductor muscles. After complete marginal resection, an excisional biopsy was performed to confirm the diagnosis.

**Interventions:**

Both patients underwent complete marginal resection without chemotherapy or radiotherapy.

**Outcomes:**

A biopsy showed a 20 × 17 × 7 cm well-differentiated, well-encapsulated liposarcoma in the 44-year-old man and a 30 × 17 × 10 cm well-differentiated liposarcoma in the 80-year-old man. These patients have achieved approximately 61 and 44 months of recurrence-free survival to date, respectively.

**Lessons:**

Here we described the long-term outcomes of 2 patients with a huge deep-seated liposarcoma in the lower extremity. Complete marginal excision of well-differentiated liposarcoma can achieve excellent recurrence-free survival.

## 1. Introduction

Liposarcomas are uncommon malignant tumors that develop in fatty tissues and feature a number of adipocytic differentiations.^[1]^ Well-differentiated, dedifferentiated, myxoid, and pleomorphic subtypes comprise liposarcoma. As for well- and de-differentiated liposarcoma, the retroperitoneum, trunk, and extremities are commonly reported sites.^[1,2]^ Unlike well-differentiated liposarcoma, which rarely metastasizes, dedifferentiated liposarcoma is aggressive with nearly 6 times higher morbidity and mortality rates. Myxoid liposarcoma, however, is most commonly found in the thigh and metastasizes to various regions. Pleomorphic liposarcoma, a high-grade undifferentiated sarcoma, is the rarest and most aggressive liposarcoma type. They are known to have frequent distant metastases and are usually found in the extremities, trunk, or retroperitoneum.

Long-term follow-up results of extremely huge liposarcomas in the submuscular layer of the thigh have rarely been reported to date. Here we share the clinical course and long-term outcomes of 2 patients with a huge deep-seated liposarcoma in the thigh.

## 2. Case presentation

Two cases of deep-seated liposarcoma in the thigh were treated at our institution over the last 10 years.

**Figure 1.**
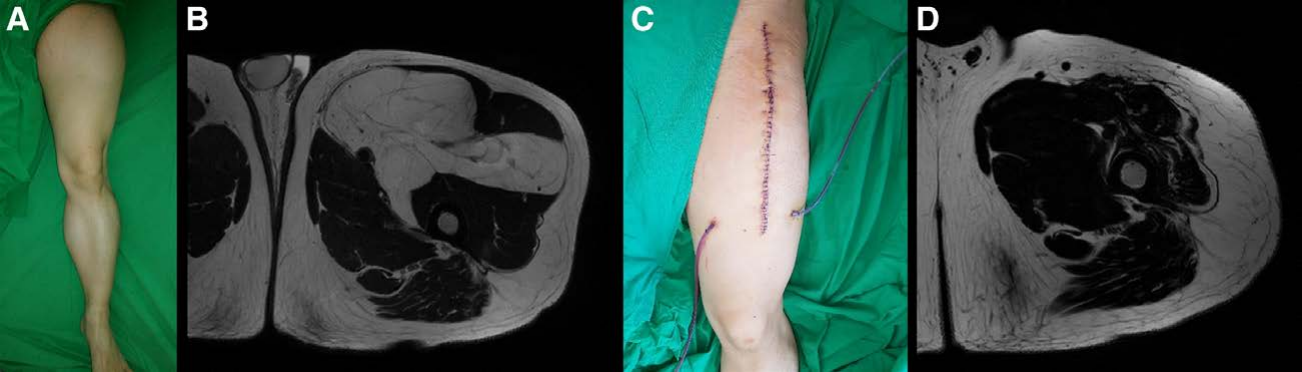
Case 1. M/44. (A) Preoperative clinical photo of the left leg. (B) Preoperative MRI, axial view. (C) Postoperative clinical photo of the left thigh. (D) Postoperative MRI, axial view. MRI, magnetic resonance imaging.

**Figure 2.**
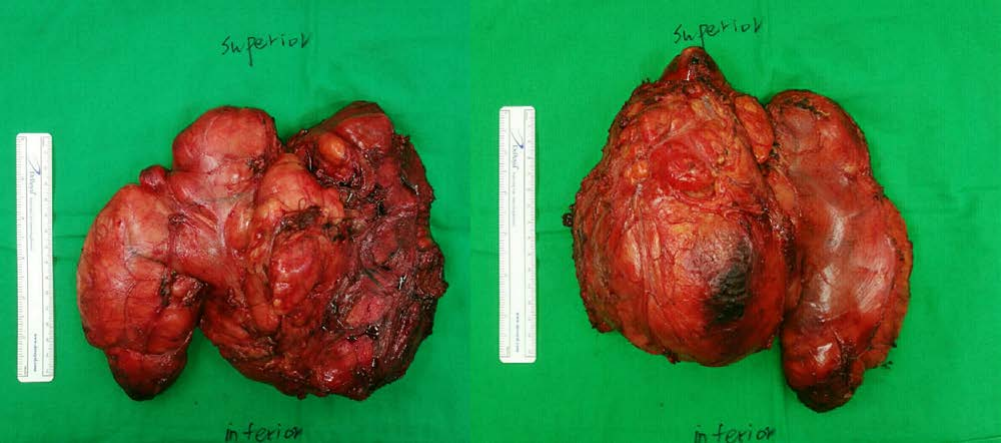
Resected liposarcoma mass of case 1.

First, a 44-year-old man visited our outpatient clinic presenting with a left thigh mass (Fig. 1A). Magnetic resonance imaging (MRI) showed a 14 × 8 × 21 cm well-differentiated liposarcoma between the sartorius and iliopsoas muscles, part of which lay near a major neurovascular bundle (Fig. 1B). A preoperative workup showed no evidence of distant metastasis. A marginal local excision was performed, the entire capsule of the mass was preserved, and perivascular fat encircling the femoral neurovascular bundle was dissected and removed en bloc (Fig. 1C and D). Sacrificing branches of the motor nerve penetrating the mass was inevitable. The sartorius muscle was cut to approach the inner-thigh mass and repaired after the resection. The resected mass was a 20 × 17 × 7 cm well-differentiated well-encapsulated liposarcoma weighing 1289 g without lymphovascular invasion (Fig. 2).

**Figure 3.**
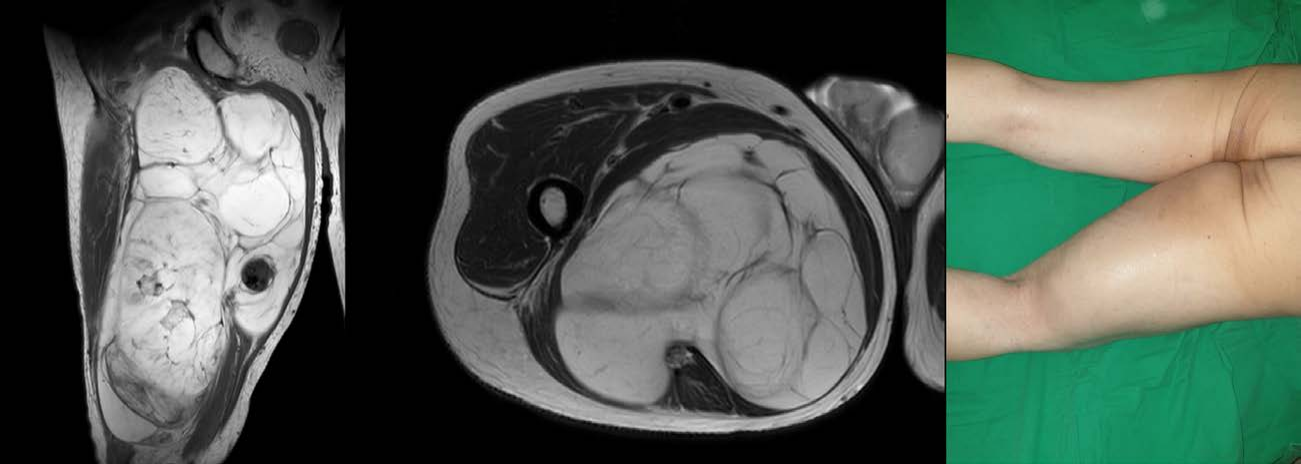
Case 2. M/80. Preoperative magnetic resonance image and clinical photo of the thigh.

**Figure 4.**
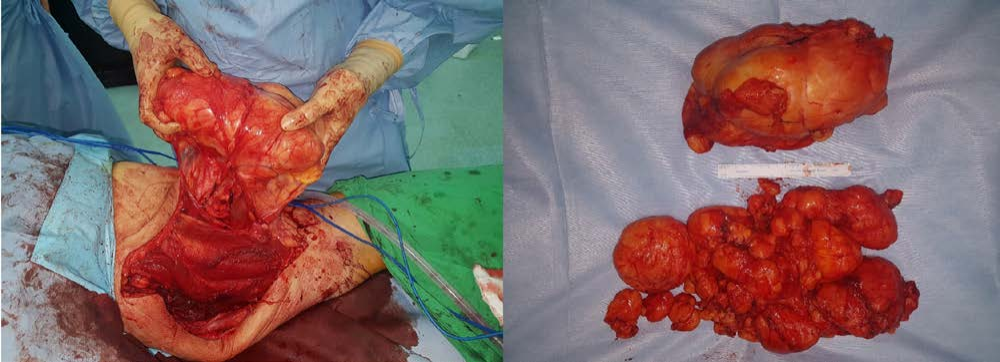
Case 2 intraoperative photo of the mass and resected liposarcoma mass.

An 80-year-old man visited our outpatient clinic presenting with a right posterior thigh mass (Fig. 3). MRI showed a 14 × 12 × 31.5 cm lipomatous mass in the posterior compartment of the right thigh mainly involving the right adductor muscles (Fig. 3). Complete surgical resection with local flap coverage was performed (Fig. 4). Only the vessels feeding the mass were dissected and ligated. The resected mass was ultimately identified as a well-differentiated liposarcoma weighing 3.4 kg and 30 × 17 × 10 cm in size.

As the tumor lengths were >15 cm in the greatest dimension without lymph node metastasis, both were confirmed as Stage IIIB T4 N0 by American Joint Committee on Cancer 8th edition criteria. There was no evidence of recurrence, and no complications were noted in the 61- and 44-month follow-up periods, respectively. Further chemotherapy or radiotherapy was not required in either case.

## 3. Discussion

Unlike other sarcomas, liposarcoma subtypes and grades can be diagnosed by radiologic imaging owing to the presence of macroscopic fat. Computed tomography (CT) and MRI can be used to characterize liposarcomas. Although CT scans are almost as efficient as MRI in terms of diagnosis, MRI is superior in determining the presence of neurovascular or muscular invasion.^[3]^ To preoperatively predict postoperative morbidities regarding surrounding neurovascular or muscular structures, we chose MRI over CT as the diagnostic tool.^[4]^ As subtype-specific treatment is of utmost importance for liposarcoma, a preoperative image-guided biopsy would be a fine option for diagnosis.^[5]^ However, we decided to perform an excisional biopsy without a preoperative biopsy as MRI was already performed considering the possible cancer dissemination following the biopsy.

Well-differentiated liposarcomas, which account for nearly half of all liposarcomas, have minimal metastatic potential, unlike other subtypes. Well-differentiated liposarcomas are insensitive to both radiotherapy and chemotherapy.^[1]^ Thus, surgical resection is perhaps the only and most critical treatment. The appropriate surgical margin for well-differentiated liposarcoma remains under debate; some claim wide excision as the treatment of choice due to possible local recurrence, while others believe marginal excision is sufficient due to scarce metastatic potential.^[2,6]^ Well-differentiated liposarcoma recurs locally at a rate of around 10%; however, it is well-managed by repeated excision of the recurrent mass without major morbidity.^[2]^ As both of our patients were preoperatively evaluated as having stage IIIB disease, the postoperative prognosis was expected to be good, with only possible local recurrence without distant metastasis.

Considering the expected prognosis, we chose complete marginal excision as the treatment of choice. Both masses involved the muscles and vessels, parts of which required sacrifice during the resection. The patient whose motor nerve branch was resected suffered from left lower leg motor weakness and a mild gait disturbance postoperatively, but this did not affect his daily life. After serial rehabilitation training focusing mainly on knee extension, his symptoms significantly improved. The second patient did not complain of any functional disability. There has been no evidence of any local or distant relapse to date in either case and despite the inevitable sacrifice of the muscle and nerves, the perioperative morbidity was minimal.

## 4. Conclusions

The cases introduced here showed recurrence-free survival of approximately 61 and 44 months to date, respectively. Although challenging, complete marginal resection of liposarcoma while preserving other adjacent structures of deep-seated liposarcoma whenever possible would lead to an outstanding long-term result with great functional outcomes.

## Author contributions

**Conceptualization:** Seong Oh Park.

**Data curation:** Hyung Woo Wang.

**Formal analysis:** Hyung Woo Wang.

**Investigation:** Hyung Woo Wang.**Supervision:** Seong Oh Park, Youn Hwan Kim.

**Visualization:** Youn Hwan Kim.

**Writing – original draft:** Hyung Woo Wang.

**Writing – review & editing:** Seong Oh Park, Youn Hwan Kim.

## References

[1] Lee ATJ Thway K Huang PH. Clinical and molecular spectrum of liposarcoma. J Clin Oncol. 2018;36:151–9. 29220294 10.1200/JCO.2017.74.9598 PMC5759315

[2] Choi KY Jost E Mack L. Surgical management of truncal and extremities atypical lipomatous tumors/well-differentiated liposarcoma: a systematic review of the literature. Am J Surg. 2020;219:823–7. 32029218 10.1016/j.amjsurg.2020.01.046

[3] Vijay A Ram L. Retroperitoneal liposarcoma: a comprehensive review. Am J Clin Oncol. 2015;38:213–9. 24136142 10.1097/COC.0b013e31829b5667

[4] Le Nail LR Crenn V Rosset P. Management of adipose tumors in the limbs. Orthop Traumatol Surg Res. 2022;108:103162. 34863958 10.1016/j.otsr.2021.103162

[5] Ikoma N Torres KE Somaiah N. Accuracy of preoperative percutaneous biopsy for the diagnosis of retroperitoneal liposarcoma subtypes. Ann Surg Oncol. 2015;22:1068–72. 25354575 10.1245/s10434-014-4210-8 PMC4520392

[6] Thway K. Well-differentiated liposarcoma and dedifferentiated liposarcoma: an updated review. Semin Diagn Pathol. 2019;36:112–21. 30852045 10.1053/j.semdp.2019.02.006

